# Phylogenomic analysis of aquatic and clinical OXA-23-positive *Acinetobacter baumannii* belonging to the international clone 5 (ST79) from Southeast Brazil^☆^

**DOI:** 10.1016/j.onehlt.2025.101140

**Published:** 2025-07-11

**Authors:** Thais Martins-Gonçalves, Elder Sano, Gregory Melocco, Karine Dantas, Fernanda Esposito, Johana Becerra, Herrison Fontana, Gustavo Queiroga, Jesus G.M. Pariona, Rodrigo Cayô, Ana C. Gales, Maria I.Z. Sato, Nilton Lincopan

**Affiliations:** a Department of Microbiology, Institute of Biomedical Sciences, University of São Paulo (USP), São Paulo, Brazil; b One Health Brazilian Resistance Project (OneBR), Brazil; c Antimicrobial Resistance Institute of São Paulo (ARIES), São Paulo, Brazil; d Department of Clinical and Toxicological Analysis, School of Pharmacy, University of São Paulo (USP), São Paulo, Brazil; e Department of Biological Science, Institute of Environmental, Chemical and Pharmaceutical Sciences (ICAQF), Federal University of São Paulo (UNIFESP), Diadema, São Paulo, Brazil; f Department of Internal Medicine, Division of Infectious Diseases, Federal University of São Paulo (UNIFESP), São Paulo, Brazil; g Environmental Company of São Paulo State (CETESB), São Paulo, Brazil

**Keywords:** Antimicrobial resistance, Critical priority pathogen, Carbapenemase, Microbial aquatic contaminants, Genomic surveillance

## Abstract

Categorized as a critical priority pathogen by the World Health Organization (WHO), carbapenem-resistant *Acinetobacter baumannii* (CRAB) is a leading cause of healthcare-associated infections (HAIs) worldwide. Although the environmental spread of healthcare-acquired *A. baumannii* strains through hospital wastewater and urban sewage has been suggested, evidence from genomic studies is still lacking. In this study, as part of the Grand Challenges Explorations: New Approaches to Characterize the Global Burden of Antimicrobial Resistance Program, we present genomic data supporting the expansion of international clone IC5/ST79 (Pasteur) to an anthropogenically impacted urban river in Brazil. The environmental OXA-23-producing *A. baumannii* Ab120 strain conserved a broad resistome to clinically important antimicrobials and carried efflux pump genes associated with tolerance to disinfectants. On the other hand, a wide virulome associated with colonization, evasion of the immune system, as well as survival and persistence in distinct environments was predicted. Noteworthy, a comparative phylogenomic analysis of the environmental Ab120 strain provided evidence that indicates its nosocomial origin and genomic relationship with human-derived OXA-23-positive clinical strains isolated between 2010 and 2017 in Brazil. Our genomic findings suggest adaptation of OXA-23-producing CRAB ST79 beyond hospital settings into non-clinical environments, which is a critical issue deserving active surveillance, particularly in Pan-American countries with a currently endemic clinical status.

## Highlights

• OXA-23-producing *A. baumannii* IC5 (ST79) was recovered from a polluted urban river.
• Environmental ST79 shared similar resistome and virulome with clinical lineages.
• Phylogenomic analysis highlighted clonal relationship with clinical endemic strains.
• Expansion of carbapenem-resistant *A. baumannii* ST79 to impacted river is discussed.

## Graphical abstract

**Figure:**
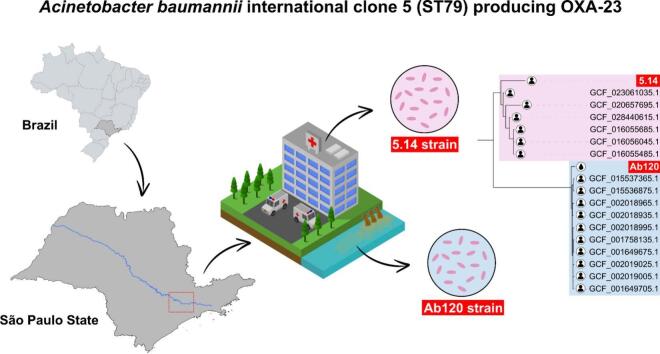

## 1 Introduction

The rapid dissemination of carbapenem-resistant *Acinetobacter baumannii* (CRAB) strains is a global public health issue [1], frequently associated with high morbimortality rates, especially among hospitalized patients [2]. In this regard, the World Health Organization (WHO) updated the bacterial priority pathogens list, confirming CRAB as a member of the critical priority group [3]. The endemic status of CRAB strains has been mainly associated with the production of OXA-23-type carbapenemases by international clones (IC) [4]. Currently, in Pan-American countries, IC5 is one of the predominant lineages in clinical settings, with sequence type 79 (ST79) being reported across Argentina, Bolivia, Brazil, Canada, Chile, Colombia, Ecuador, Honduras, Mexico, Panama, Paraguay, Peru, the United States, Uruguay, and Venezuela [1,[5], [6], [7], [8], [9], [10], [11], [12], [13], [14]]. Worryingly, CRAB strains have expanded beyond hospital settings into non-clinical environments. In fact, several countries have documented OXA-23-producing *A. baumannii* strains in hospital wastewaters [[15], [16], [17], [18], [19], [20]], wastewater treatment plants [18,21,22], and natural water bodies, such as rivers [23,24]. However, genomic and related environmental data remain scarce and are extremely valuable, since the presence of healthcare-associated clones in aquatic environments represents a risk to the interdependent human-animal-environment interface [[25], [26], [27]]. Herein, we performed a comparative genomic investigation of an environmental OXA-23-producing *A. baumannii* ST79 strain recovered from a polluted river with clinical strains, in order to elucidate the background spillover of the dissemination of CRAB beyond hospital walls into nature.

## 2 Materials and methods

### 2.1 Sample collection and bacterial isolation

During a Brazilian surveillance study (OneBR project) conducted to monitor the presence of multidrug-resistant (MDR) bacteria in anthropogenically impacted aquatic environments in Southeastern Brazil, 39 surface water samples were collected and provided by the Environmental Company of São Paulo State (CETESB), from ten locations along the Tietê and Pinheiros Rivers. The Tietê River is the main river in São Paulo State, running approximately 1100 km from east to west through the state, while the Pinheiros River is a tributary of the Tietê River that runs 25 km across São Paulo city [24]. The Tietê River is divided into the Upper, Middle, and Lower Tietê. This division is based on the physiography of the state of São Paulo. Samples were collected from the Upper Tietê, where the river receives most of the effluents and diffuse wastes from the São Paulo metropolis, and from the less polluted Middle and Lower Tietê River stretches (Fig. S1). As previously described [24], surface water samples (500 mL) were collected in sterile bottles. Subsequently, 100 mL of each sample was filtered through sterile membrane filters (0.45 μm pore size). The membrane filters were placed on MacConkey agar plates and incubated at 37 °C for 24 h. Next, the filters were transferred to sterile tubes with 10 mL of Mueller–Hinton broth. After vortexing, a 100 μL aliquot of each culture was streaked onto MacConkey agar plates supplemented with imipenem (MC + IPM) and further incubated at 37 °C for 24 h. Colonies presenting different morphotypes were streaked into MC + IPM to obtain pure bacterial cultures. Bacterial identification was performed by matrix assisted laser desorption/ionization time-of-flight mass spectrometry (MALDI-TOF, Bruker MALDI Biotyper CA System) [28]. In this regard, three OXA-23-producing *A. baumannii* strains (Ab105, Ab120, and Ab150) were isolated from different locations (*n* = 3/10) along the Tietê River (TIET-04180/Ab105 strain: location 5, S 23° 31′ 18″, W 46° 37′ 52″; TIET-04900/Ab120b strain: location 3, S 23° 27′ 16″, W 46° 54′ 36″) and Pinheiros River (PINH-04900/Ab150 strain: location 8, S 23° 31′ 52″, W 46° 44′ 54″) (Fig. S1). These strains were classified as clonally related by enterobacterial repetitive intergenic consensus-polymerase chain reaction (ERIC-PCR) [29]. The Ab120 strain, recovered in 2010 [24], was chosen as a representative isolate for whole genome sequence (WGS) investigation. Additionally, a clinical OXA-23-positive *A. baumannii* strain (5.14), representative of the ST79 clone circulating in Brazilian hospitals from 2008 [7], recovered from a tertiary hospital whose treated sewage effluent is released into the Tietê River, was also sequenced for comparative genomic analysis.

### 2.2 Antimicrobial susceptibility testing

Antimicrobial susceptibility tests were performed by disk diffusion and/or broth microdilution methods. The following antimicrobials were tested: amikacin, ampicillin/sulbactam, cefepime, cefiderocol, cefotaxime, ceftazidime, ceftriaxone, ciprofloxacin, colistin, doxycycline, gentamicin, imipenem, levofloxacin, meropenem, minocycline, piperacillin/tazobactam, tetracycline, tobramycin, and trimethoprim/sulfamethoxazole. Results were interpreted according to the Clinical and Laboratory Standards Institute (CLSI) and the European Committee on Antimicrobial Susceptibility Testing (EUCAST) guidelines [30,31].

### 2.3 Whole genome sequence analysis

Genomic DNA of Ab120 and 5.14 strains was extracted using PureLink Quick Gel Extraction & PCR Purification Combo Kit (Life Technologies, Carlsbad, CA). Genomic library construction was performed using a 2 × 75-bp paired-end Nextera XT DNA Library Preparation Kit (Illumina Inc., Cambridge, UK) and, subsequently, sequenced on the NextSeq550 platform (Illumina), according to the manufacturer's guidelines. Raw sequencing data with a PHRED quality score below 20 were removed using TrimGalore v.0.6.5 (https://github.com/FelixKrueger/TrimGalore), whereas de novo genome assembly was performed using Unicycler v.0.4.8 (https://github.com/rrwick/Unicycler) [32]. Genomes sequences were annotated using NCBI Prokaryotic Genome Annotation Pipeline (PGAP) v.3.2 (http://www.ncbi.nlm.nih.gov/genome/annotation_prok/) [33], and Rapid Annotation System Technology (RAST) pipeline (https://github.com/MG-RAST). Multilocus sequence typing (MLST) prediction was performed using MLST v.2.0 (https://cge.cbs.dtu.dk/services/MLST/) [34]. Resistome was predicted using ResFinder (https://cge.food.dtu.dk/services/ResFinder/) and the Comprehensive Antibiotic Resistance Database (CARD) (https://card.mcmaster.ca/) [35,36]. Virulome was predicted by using *A. baumannii* typing database (https://pubmlst.org/bigsdb?db=pubmlst_abaumannii_seqdef) [37], Virulence Factor Database (VFDB) (https://github.com/haruosuz/vfdb) [38], and VFanalyzer (http://www.mgc.ac.cn/cgi-bin/VFs/v5/main.cgi) [39]. Capsular polysaccharide (KL) and outer core of lipooligosaccharide (OCL) were predicted using Kaptive (https://kaptive-web.erc.monash.edu/) [40,41]. For all predicted genes, a ≥ 95 % identity threshold was used for gene prediction.

### 2.4 Phylogenomic analysis of *A. baumannii* ST79 strains

For comparative genomic analysis of environmental Ab120 and clinical 5.14 strains with global *A. baumannii* ST79 genomes, we retrieved all *A. baumannii* strains (NCBI:txid470) from NCBI RefSeq, and identified their STs using MLST v.2.23.0 (https://github.com/tseemann/mlst), selecting all ST79 strains (*n* = 141) [42]. NCBI BioSamples data were retrieved using the “biosample2table” script (https://github.com/stajichlab/biosample_metadata). ABRicate v.1.0.1 (https://github.com/tseemann/abricate) was used with ResFinder database to identify antimicrobial resistance genes. Prokka v.1.14.6 (https://github.com/tseemann/prokka) was used to annotate all genomes [43], and Roary v.3.13.0 (https://github.com/sanger-pathogens/Roary) was used to identify core genes and align core genomes [44]. SNP-sites v.2.5.1 (https://github.com/sanger-pathogens/snp-sites) was used to call SNP within aligned core genomes [45], and SNP-dists v0.8.2 (https://github.com/tseemann/snp-dists) was used to generate a SNP distance matrix. FastTree v.2.1.11 (https://www.microbesonline.org/fasttree) was used to generate a SNP-based approximately-maximum-likelihood phylogenetic tree [46]. The tree was then midpoint-rooted using iTOL v.6 (https://itol.embl.de) [47], which was also used to exclude a distant clade with 58 strains, and to annotate the tree with data from the National Center for Biotechnology Information (NCBI) BioSamples and ABRicate.

### 2.5 Genetic context analysis of *bla*_OXA-23_

The *bla*_OXA-23_ gene was detected and aligned by BLASTn against transposons Tn*2006* (GenBank accession number: JN107991.2), Tn*2007* (GenBank accession number: EF059914.1), Tn*2008* (GenBank accession number: KP780408.1), Tn*2008B* (GenBank accession number: CP085788.1), Tn*2008VAR* (GenBank accession number: LT594095), Tn*2009* (GenBank accession number: NZ_KM922672.1), Tn*6549* (GenBank accession number: CP009257), and AbaR4 (GenBank accession number: KJ493819). Genetic context analysis of the *bla*_OXA-23_ gene of both Ab120 and 5.14 strains were manually curated using Geneious Prime version 2022.1.1 and BLASTn algorithm (Biomatters, New Zealand).

## 3 Results and discussion

As an opportunistic pathogen, *A. baumannii* is considered a leading cause of healthcare-associated infections worldwide. In this regard, although an increased number of genomic studies have been conducted in the last years, most of them have focused on hospital isolates alone [1,10,48]. More recently, evidence on the dissemination of CRAB beyond hospital settings has led to consider this pathogen as a One Health problem, which could have important public health implications in terms of their pathogenic and antibiotic resistance potential [[49], [50], [51], [52]]. Therefore, sequencing CRAB strains from human and non-human sources is crucial to understand dissemination dynamics of international high-risk lineages, as well as other emerging clones [10,50].

In this study, we conducted a genomic investigation of an environmental OXA-23-producing *A. baumannii* ST79 strain recovered from an anthropogenically impacted urban river, and a comparative phylogenomic analysis with nosocomial strains belonging to ST79, to elucidate the dissemination of CRAB beyond hospital walls to non-clinical environments. In this regard, the environmental Ab120 strain shared a similar MDR profile (i.e., broad-spectrum cephalosporins, carbapenems, aminoglycosides, fluoroquinolones, and sulfamethoxazole-trimethoprim) [53] with the clinical 5.14 strain, which is representative of the ST79 clone circulating in different clinical settings since 2008, previously isolated from a tertiary Brazilian hospital, whose treated effluent is released into the Tietê River [7] (Table 1). Additionally, both strains remained susceptible to cefiderocol, colistin, doxycycline, minocycline, tetracycline, and tobramycin, while only the environmental strain was also susceptible to ampicillin/sulbactam. Genomic data of environmental Ab120 and clinical 5.14 *A. baumannii* strains (GenBank accession numbers: JAQBHQ000000000 and JAQBHP000000000) are summarized in Table 1.

**Table 1:** Genomic and microbiological characteristics of environmental and clinical carbapenem-resistant *A. baumannii* strains carrying *bla*_OXA-23_.

Characteristics	*Acinetobacter baumannii*	
Strain	Ab120	5.14
Source	Urban river water	Human blood
Date (year)	2010	2008
MLST^a^ (Pasteur/Oxford)	ST79/ST227	ST79/ST783
Outer core locus	OCL10	OCL10
K-locus	KL3	KL33

Resistance profile^b^		
β-lactams	TZP, CAZ, CTX, CRO, FEP, IPM, MEM	SAM, TZP, CAZ, CTX, CRO, FEP, IPM, MEM
Aminoglycosides	AMK, GEN, TOB	AMK, GEN, TOB
Fluoroquinolones	CIP, LVX	CIP, LVX
Folate pathway antagonists	SXT	SXT

Resistome		
β-lactams	*bla*_OXA-23_, *bla*_OXA-65_, *bla*_TEM-1__A_, *bla*_ADC-182_	*bla*_OXA-23_, *bla*_OXA-65_, *bla*_TEM-1__A_, *bla*_ADC-182_
Aminoglycosides	*aph(3′)-*VIa, *aph(6)-Id*, *aph(3″)-Ib*	*aph(3′)-*VIa, *aph(6)-Id*, *aph(3″)-Ib*
Fluoroquinolones (mutations)	GyrA (S81L), ParC (V104I, D105E)	GyrA (S81L), ParC (V104I, D105E)
Phenicols	*floR*	*floR*
Trimethoprim	*dfrA1*	*dfrA1*
Macrolides	*–*	*msr(E)*, *mph(E)*
Sulfonamides	*sul1*	*sul1*

Virulome		
Adherence	*fimTUV*, *gspO*/*pilD*, *pilABEFGHIMNOPQRSTUVWX*, *pilY1*, *tsaP*	*ata*, *fimTUV, gspO/pilD, pilABCEFGHIJMNOPQRSTUVWX*, *pilY1, tsaP*
Biofilm formation	*bfmRS*, *csuA/B, csuABCDE, pgaABCD*	*bfmRS*, *csuA/B, csuABCDE, pgaABCD*
Immune evasion	*lpsB*, *lpxABCDLM, ompA, pbpG*	*lpsB*, *lpxABCDLM, ompA*, *pbpG*
Iron uptake	*barAB*, *basABFGHIJ, bauABCDEF*, *entE*, *hemO*	*barAB*, *basABCDFGHIJ, bauABCDEF, entE*
Cell invasion	*plc2*, *plcD*	*plc1*, *plc2*, *plcD*
Oxidative stress detoxification	*katE*, *katG*	*katE*, *katG*
Disinfectants	*amvA, abeM*	*amvA, abeM*
GenBank accession number	JAQBHQ000000000	JAQBHP000000000

a MLST, multilocus sequence typing; ST, sequence type.

b AMK, amikacin; CAZ, ceftazidime; CIP, ciprofloxacin; CRO, ceftriaxone; CTX, cefotaxime; FEP, cefepime; GEN, gentamicin; IPM, imipenem; LVX, levofloxacin; MEM, meropenem; SAM, ampicillin/sulbactam; SXT, trimethoprim/sulfamethoxazole; TOB, tobramycin; TZP, piperacillin/tazobactam.

**Fig. 1:**
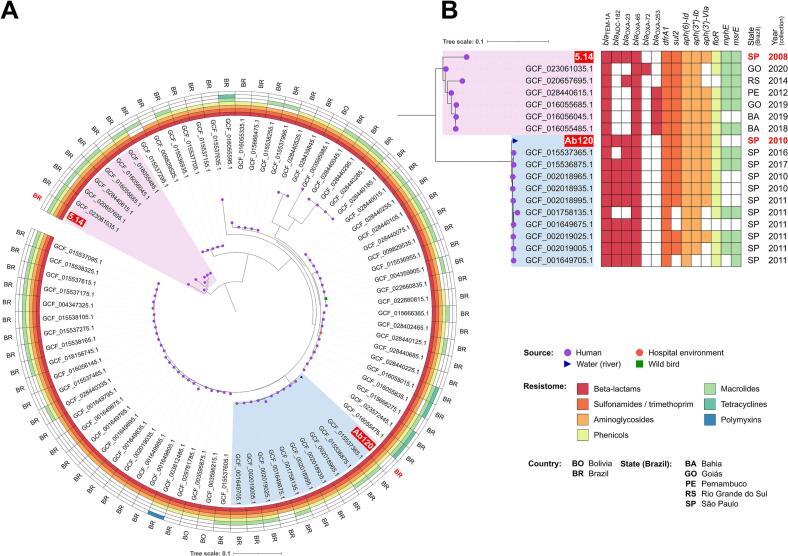
In A, a phylogenetic tree with 80 *A. baumannii* ST79 strains, their sources of isolation, the presence/absence of antimicrobial resistance genes to different antimicrobial classes, and country of origin. In B, highlighted clades in A, showing their resistome, Brazilian state, and year of collection. Tree scale: SNPs per SNP site.

Resistance-encoding genes to β-lactams (*bla*_OXA-23_, *bla*_OXA-65_, *bla*_TEM-1__A_, *bla*_ADC-182_), aminoglycosides [*aph(3′)-*VIa, *aph(6)-Id*, *aph(3″)-Ib*], sulfonamides (*sul2*), trimethoprim (*dfrA1*), and phenicols (*floR*) were predicted in both bacterial isolates, in accordance with the phenotypic resistance profile (Table 1; Table S1; Fig. 1B). In addition, point mutations related to fluoroquinolone resistance and efflux pump genes associated with tolerance to disinfectants were also predicted in both strains (Table 1).

The evaluation of the genetic context of the *bla*_OXA-23_ gene demonstrated that the IS*Aba1*-*bla*_OXA-23_-ATPase sequence array, identified in both Ab120 and 5.14 strains, was located within the Tn*2008* transposon, which is known for carrying this carbapenemase-encoding gene [54]. The insertion of the IS*Aba1* in the opposite transcriptional direction upstream of *bla*_OXA-23_ provides a strong promoter responsible for the over-expression of the oxacillinase, resulting in high minimum inhibitory concentrations (MIC) for carbapenems [55,56]. Furthermore, alignment analysis showed that Tn*2008* from environmental Ab120 and clinical 5.14 strains exhibited high similarity with the Tn*2008* transposon carried by a clinical *A. baumannii* strain isolated in Australia (GenBank accession number: KP780408.1) (Fig. S2).

Virulome analysis revealed that Ab120 and 5.14 carried several virulence-encoding genes associated with adherence, cell invasion, biofilm formation, immune modulation, iron uptake, and oxidative stress detoxification (Table 1; Table S1). In general, the wide diversity of virulence factors identified in both strains may provide them the capacity to colonize, evade immune responses, and subsequently establish infections, as well as successfully survive and persist in distinct environments [57].

MLST analysis, based on the Pasteur scheme, confirmed these isolates as belonging to the international high-risk clone ST79, endemic in Latin America [1,[8], [9], [10]], and also reported in Africa [14], Asia [14], North America, and Europe [1,10]. Additionally, MLST analysis based on the Oxford scheme revealed that Ab120 (ST79^Pas^) and 5.14 (ST79^Pas^) strains belonged to ST227 and ST783, respectively. Such MLST discrepancy was previously reported and attributed to a variation observed in the *gpi* allele [58,59]. This gene is located near the capsule biosynthesis gene cluster, where recombination replacements are common [58]. Accordingly, different K-locus in environmental Ab120 and clinical 5.14 strains were predicted (Table 1; Table S1).

Noteworthy, phylogenomic analysis based on SNPs of 1961 core genes revealed that environmental Ab120 (ST79^Pas^/ST227^Oxf^) and clinical 5.14 (ST79^Pas^/ST783^Oxf^) *A. baumannii* strains were distant from each other (3043 SNP differences) (Table S2), and while strain 5.14 grouped with other *A. baumannii* strains belonging to ST783^Oxf^ and ST1283^Oxf^ (886 to 1437 SNP differences, bootstrap = 1, BAPS cluster 2), the environmental strain Ab120 was genomically related (168 to 311 SNP differences, bootstrap = 1, BAPS cluster 1) to hospital-acquired *A. baumannii* strains recovered from two Brazilian medical centers, between 2010 and 2017 (Fig. 1B; Table S2). In fact, the potential of ST79 to remain for long periods in the hospital environment has been previously documented [9,60] (Table S2).

All ST79 *A. baumannii* genome assemblies included in the phylogenomic analysis were from Brazil (*n* = 137) and Bolivia (*n* = 3). While 139 of these strains were originated from human sources, one was recovered from the hospital environment in Northeastern Brazil in 2014 [61], and another (GCF_022660835.1), was isolated from a migratory bird in São Paulo in 2012 [51]. These data highlight the dispersion of *A. baumannii* ST79 across Brazil and South America, as well as its spread through human-animal-environment interfaces, a typical One Health issue.

In Latin America, CRAB strains belonging to ST79 have been predominant within the IC5 clade, being characterized by the production of either OXA-23 or OXA-72 carbapenemases [1]. Currently, OXA-23-producing *A. baumannii* ST79 clones are considered endemic and responsible for numerous hospital outbreaks in Brazil [[5], [6], [7],^9^,^11^], being also reported in Argentina, Canada, Chile, Colombia, Ecuador, Israel, Malaysia, Paraguay, Romania, the United States, Uruguay, South Africa, and Venezuela [1,8,10,14]. Interestingly, ST79 clones have also been associated with higher mortality rates compared to ST1, ST15, and ST25 *A. baumannii* strains isolated from patients in a Brazilian intensive care unit [9].

The occurrence of OXA-23-producing *A. baumannii* in aquatic urban environments is concerning, since this critical pathogen is primarily restricted to hospital settings. Strikingly, the occurrence of CRAB has also been reported in an urban river in France [23]. The OXA-23-positive B9 strain, isolated from the Seine River, was also highly antibiotic resistant and belonged to a clinically significant international lineage (IC2), often associated with nosocomial infections [23,52].

*A. baumannii* has developed remarkable adaptive mechanisms to persist in both healthcare and aquatic environments [50,52,62]. The environmental persistence of CRAB supports its role as a One Health pathogen [50,63]. In this respect, the environmental contamination with sub-inhibitory concentrations of antibiotics and biocides may contribute to the selection and successful expansion of high-risk clones beyond hospital walls [64], emphasizing the potential for urban rivers to act as reservoirs of international lineages harboring broad resistome and virulome. Some studies suggest that healthcare-associated *A. baumannii* strains could persist through wastewater treatment processes [65], being able to disseminate from hospital wastewater and urban sewage into natural aquatic environments [66,67], where they can survive for up to 50 days and potentially disseminate and/or accumulate in river sediments [62].

The occurrence of CRAB ST79 in aquatic environments has significant ecological and public health implications, including: i) potential for horizontal gene transfer; ii) long-term persistence in nature; iii) colonization of livestock, wildlife, companion animals, or humans, via contaminated drinking water or recreational activities, creating new reservoirs for CRAB; and, iv) risk of community-acquired infections, especially among immunocompromised individuals or those with direct water exposure (e.g., open wounds).

Therefore, further studies must be directed to understand the evolutionary changes of ST79 in the aquatic environment, evaluate biofilm-forming capacity on microplastics, perform proteomic analysis under environmental stressors (e.g., fluctuating temperature, pH, and salinity), investigate interactions with native aquatic microbiota, develop quantitative microbial risk assessment models to estimate the risk of human and animal exposure, and expand surveillance to wildlife inhabiting the associated aquatic ecosystem.

In summary, we present genomic evidence supporting the adaptation of the nosocomial high-risk OXA-23-producing *A. baumannii* ST79 clone into an anthropogenically impacted urban river, highlighting a One Health issue deserving active surveillance.

## CRediT authorship contribution statement

**Thais Martins-Gonçalves:** Writing – review & editing, Writing – original draft, Visualization, Validation, Methodology, Investigation, Formal analysis, Data curation, Conceptualization. **Elder Sano:** Writing – review & editing, Writing – original draft, Visualization, Validation, Methodology, Formal analysis. **Gregory Melocco:** Writing – review & editing, Writing – original draft, Visualization, Validation, Formal analysis. **Karine Dantas:** Writing – review & editing, Writing – original draft, Visualization, Validation, Formal analysis. **Fernanda Esposito:** Writing – review & editing, Writing – original draft, Visualization, Validation, Methodology, Formal analysis, Data curation. **Johana Becerra:** Writing – review & editing, Writing – original draft, Visualization, Validation, Methodology, Formal analysis. **Herrison Fontana:** Writing – review & editing, Writing – original draft, Visualization, Validation, Methodology, Investigation, Formal analysis, Data curation. **Gustavo Queiroga:** Writing – review & editing, Writing – original draft, Visualization, Validation, Methodology, Investigation, Formal analysis. **Jesus G.M. Pariona:** Writing – review & editing, Writing – original draft, Visualization, Validation, Formal analysis. **Rodrigo Cayô:** Writing – review & editing, Writing – original draft, Visualization, Validation, Methodology, Investigation, Formal analysis. **Ana C. Gales:** Writing – review & editing, Writing – original draft, Visualization, Validation, Investigation, Formal analysis. **Maria I.Z. Sato:** Writing – review & editing, Writing – original draft, Visualization, Validation, Investigation, Formal analysis, Conceptualization. **Nilton Lincopan:** Writing – review & editing, Writing – original draft, Visualization, Validation, Project administration, Investigation, Funding acquisition, Conceptualization.

## Funding

This study was supported by the 10.13039/100000865Bill and Melinda Gates Foundation (Grand Challenges Explorations Brazil OPP1193112). Under the grant conditions of the Foundation, a CC BY or equivalent license is applied to the author accepted manuscript version arising from this submission. Additionally, this study was supported by the 10.13039/501100001807Fundação de Amparo à Pesquisa do Estado de São Paulo (FAPESP, 2020/08224–9), 10.13039/501100003593Conselho Nacional de Desenvolvimento Científico e Tecnológico (CNPq, 422984/2021–3). T.M.G. is a research fellow of FAPESP (Grant 2024/20180-8). G.M. is a research fellow of CNPq (130767/2021–2). K.D. is a research fellow of FAPESP (Grant 2024/07885-2). F.E. is a research fellow of FAPESP (2019/15578–4). JB is a research fellow of FAPESP (2023/18292–0). G.Q. is a research fellow of CNPq (130028/2023–1). R.C. (307915/2022–0), A.C.G. (312066/2019–8), and N.L. (314336/2021–4) were supported by grants from the CNPq. We extend our gratitude to the FAPESP for their support through the awarded grant (Process No. 21/10599-3).

## Declaration of competing interest

A.C.G. has recently received research funding and/or consultation fees from Enthasis, Eurofarma, MSD, Pfizer, Sandoz, and United Medical. Other authors have nothing to declare. This study was not financially supported by any Diagnostic/Pharmaceutical company.

The authors declare the following financial interests/personal relationships which may be considered as potential competing interests:

Nilton Lincopan reports financial support was provided by State of Sao Paulo Research Foundation. Nilton Lincopan reports financial support was provided by National Council for Scientific and Technological Development. If there are other authors, they declare that they have no known competing financial interests or personal relationships that could have appeared to influence the work reported in this paper.

## Data Availability

The datasets presented in this study can be found in online repositories, including NCBI Datasets (https://www.ncbi.nlm.nih.gov/datasets/). Both Ab120 and 5.14 genome shotgun projects have been deposited at DDBJ/ENA/GenBank under the accession JAQBHQ000000000 and JAQBHP000000000, respectively.

## Appendix A Supplementary data

Figure S2 Supplementary material 2

Table S1. Epidemiological and clinically relevant genomic data of *Acinetobacter baumannii* IC5 (ST79) from Brazil Supplementary material 3

Table S2. SNP matrix and BAPS of global *A. baumannii* strains belonging to ST79 Supplementary material 4
