# Supplementary material for: Phylogenomic analysis of aquatic and clinical OXA-23-positive Acinetobacter baumannii belonging to the international clone 5 (ST79) from Southeast Brazil
Source: One Health. 2025 Jul 11;21:101140. doi: 10.1016/j.onehlt.2025.101140 (PMC12284700; doi:10.1016/j.onehlt.2025.101140)
Supplement: Figure S1 [file mmc1.docx]

**Supplementary Figure 1**


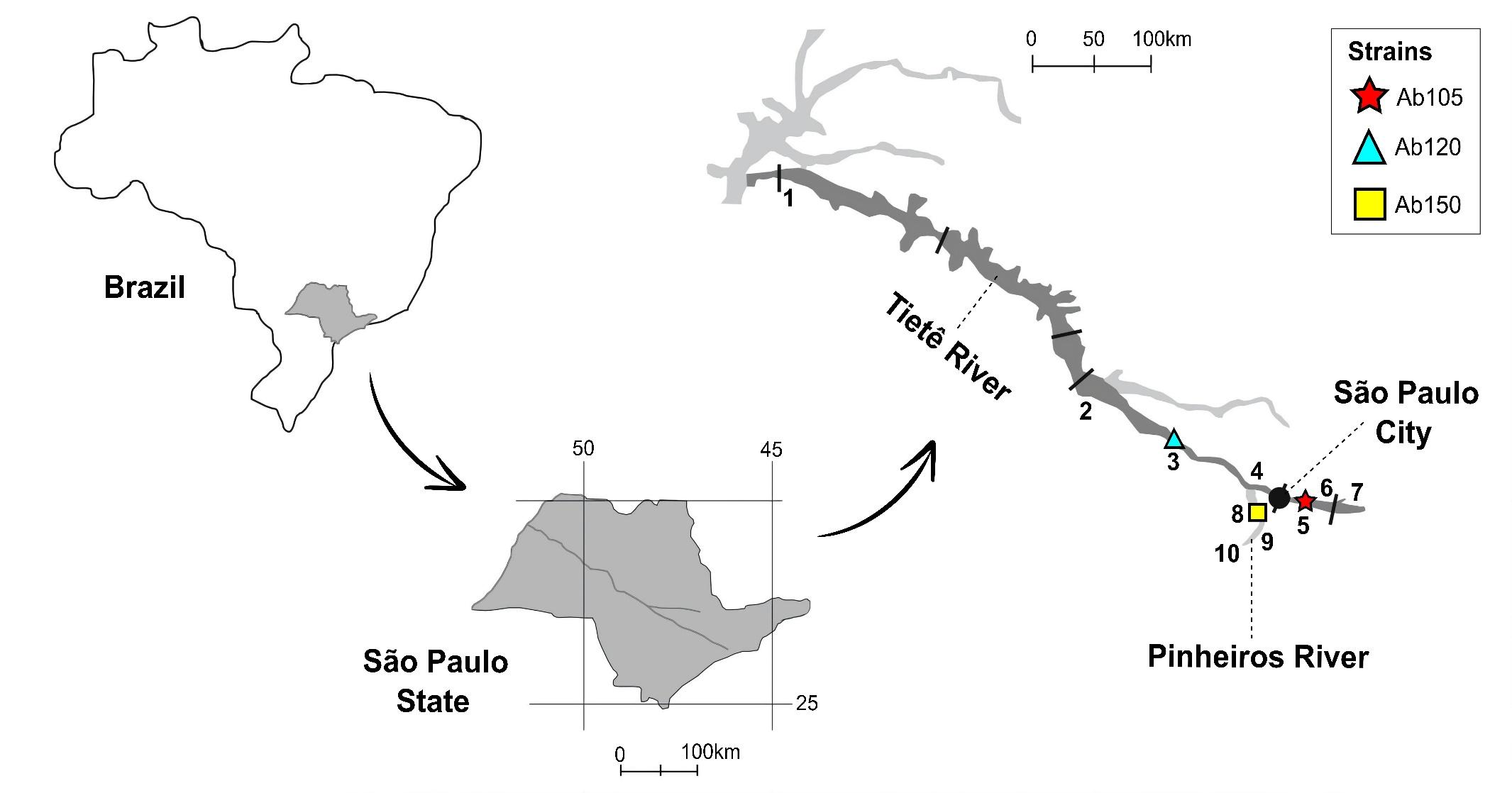
**Figure S1.** Map illustrating sampling locations along the Tietê and Pinheiros rivers in the metropolitan region of São Paulo, Brazil (Turano et al., 2016). Three OXA-23-producing *A. baumannii* ST79 strains were recovered from water samples collected at sites 3 (Ab120), 5 (Ab105), and 8 (Ab150). The representative Ab120 strain, sequenced in this study, was isolated from the Upper Tietê River (TIET-04900: S 23° 27' 16'', W 46° 54' 36'').
