# Supplementary material for: Phylogenomic analysis of aquatic and clinical OXA-23-positive Acinetobacter baumannii belonging to the international clone 5 (ST79) from Southeast Brazil
Source: One Health. 2025 Jul 11;21:101140. doi: 10.1016/j.onehlt.2025.101140 (PMC12284700; doi:10.1016/j.onehlt.2025.101140)

**Figure S2.** Overall comparison of Tn*2008* containing the *bla*_OXA-23_ gene carried by environmental (Ab120) and clinical (5.14) *A. baumannii* ST79 strains against Tn*2008* carried by the clinical 08325850 strain (GenBank: [KP780408.1](https://www.ncbi.nlm.nih.gov/nuccore/KP780408.1)). The Tn*2008* element presented 2,724 kbp and was composed of IS*Aba1* (black arrow), *bla*_OXA-23_ (blue arrow), and an ATPase (light-gray arrow).


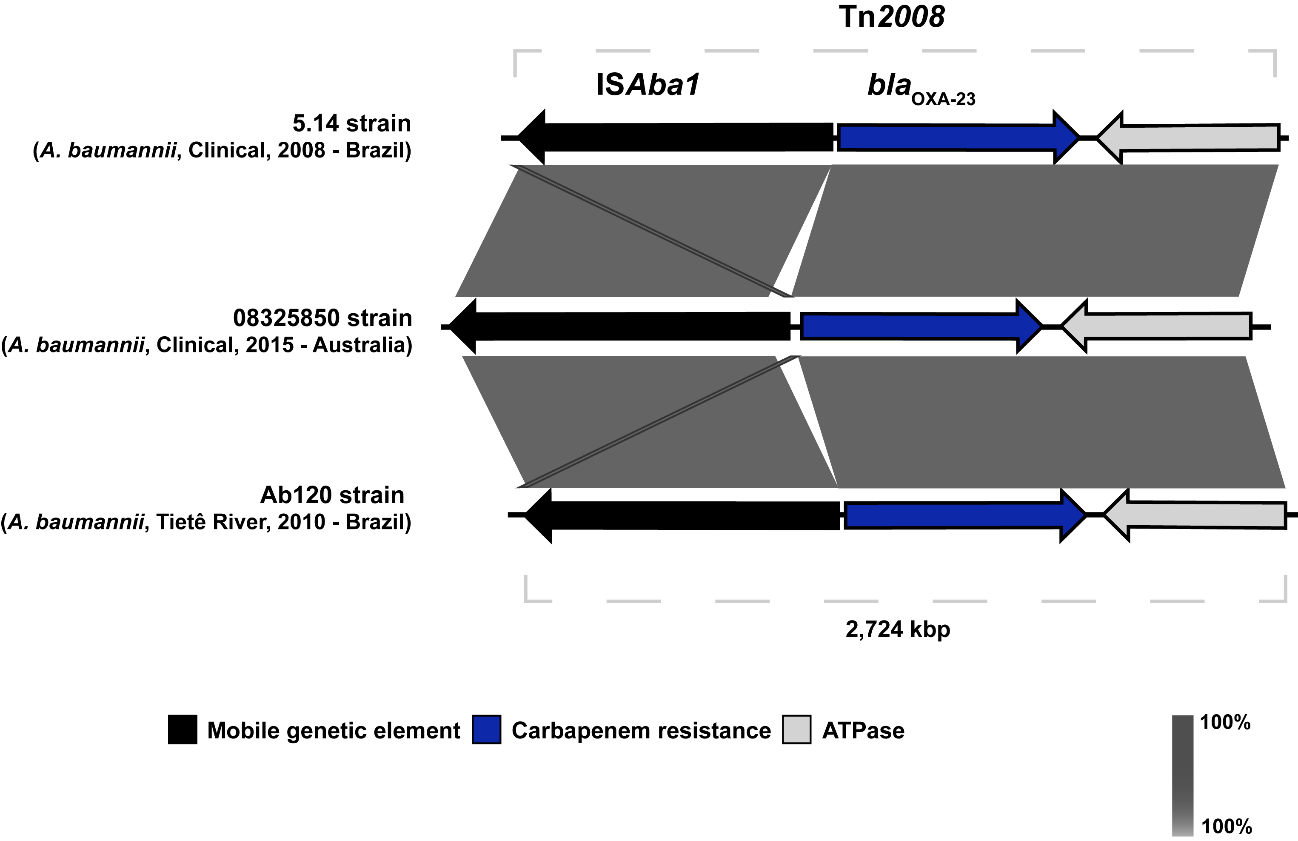

Supplement: Figure S2 [file mmc2.docx]
